# Effects of growth stage on the fermentation quality, microbial community, and metabolomic properties of Italian ryegrass (*Lolium multiflorum* Lam.) silage

**DOI:** 10.3389/fmicb.2022.1054612

**Published:** 2023-01-13

**Authors:** Zhihui Fu, Lin Sun, Zhijun Wang, Jingyi Liu, Meiling Hou, Qiang Lu, Junfeng Hao, Yushan Jia, Gentu Ge

**Affiliations:** ^1^College of Grassland, Resources and Environment, Key Laboratory of Forage Cultivation, Processing and High Efficient Utilization of Ministry of Agriculture, and Key Laboratory of Grassland Resources, Inner Mongolia Agricultural University, Ministry of Education, Hohhot, China; ^2^Inner Mongolia Academy of Agricultural & Animal Husbandry Sciences, Hohhot, China; ^3^College of Life Science, Baicheng Normal University, Baicheng, China; ^4^College of Agriculture, Ningxia University, Yinchuan, China

**Keywords:** Italian ryegrass silage, bacterial community, metabolomics profiles, SMRT, UHPLC–MS/MS

## Abstract

**Introduction:**

This study aimed to investigate the effects of different growth stages (booting period-SYK; initial flowering-SCK; full flowering-SSK) on the fermentation quality, microbial community, metabolic pathways and metabolomic characteristics of Italian ryegrass silage.

**Methods:**

Single molecule real-time (SMRT) sequencing and ultra-high performance liquid chromatography-mass spectrometry (UHPLC-MS/MS) were used to analyze bacterial communities and metabolites, respectively.

**Results:**

After 60 d of fermentation, SYK had the lowest pH and the highest lactic acid content, which were significantly different from the other groups. The bacteria with the highest abundance in SYK, SCK and SSK groups were *Lactiplantibacillus plantarum* (63.98%), Weissella minor (28.82%) and *Levilactobacillus brevis* (64.81%), respectively. In addition, among the main differential metabolites in different growth stages, the number of amino acids was the most, and the corresponding metabolic pathways were mainly amino acid metabolic pathways. The biosynthesis of phenylalanine, tyrosine and tryptophan was significantly enriched (p<0.01) at booting stage and full flowering stage. Purine metabolism and ABC transporter pathway were significantly enriched at the initial flowering (p<0.001). *Lactiplantibacillus plantarum* had a negative correlation with xanthine and ganoderic acid F. *Weissella minor* had a positive correlation with D-Mannose and ganoderic acid F. *Levilactobacillus brevis* had a positive correlation with xanthine, and *Latilactobacillus sakei* had a positive correlation with cinnamic acid, D-Mannose, 2-Hydroxycinnamic acid and uridine.

**Discussion:**

In conclusion, this study reveals the interaction mechanisms between ryegrass raw materials at different growth stages and epiphytic microorganisms during ensiling fermentation, providing new ideas for screening functional lactic acid bacteria, and laying a theoretical foundation for the production of safe and high-quality silage.

## Introduction

Ensiling is a microbial driven process and an appropriate way to preserve forages. During ensiling, microorganisms ferment water soluble carbohydrates into acids under anaerobic conditions, reducing the pH value, thereby inhibiting the reproduction of undesirable microorganisms to achieve the purpose of long-term preservation of forage nutrients (Bai et al., 2022; Li et al., 2022a). A successful silage fermentation process is affected by a variety of factors, such as the characteristics of the silage material at harvest period, climatic conditions, and the epiphytic microbial community of the silage materials (Li et al., 2022b). Long et al. (2022) reported that the growth stage was a key factor affecting the quality of silage, because growth stage affects the chemical composition and ensilability of raw materials. During forage growth, with the increase of forage maturity cell wall content is continually deposited, stem-leaf ratio and fiber content increase, and crude protein content decreases (Yin et al., 2022). In addition, the epiphytic microbiota in the aerial parts of plants vary by growth stage. During the growing season, microbial populations on leaves are initially dominated by bacteria, followed by yeasts and finally filamentous fungi (Yin et al., 2022). Clarifying the effect of epiphytic microbial population and chemical composition on the quality of silage can help to further understand the mechanism of ensiling fermentation.

Due to its high yield, digestibility, and nutritional value, especially highly soluble and degradable nitrogen and carbohydrates, Italian ryegrass (*Lolium multiflorum* Lam.) is widely used for silage making in temperate areas (Stergiadis et al., 2015; Wang et al., 2022b). During fermentation, regular changes in the microbial community occur during the aerobic and anaerobic stages, and studying these changes is crucial for improving silage quality (You et al., 2022). With the development of microbial sequencing technology, PacBio SMRT sequencing technology has been widely used in microbiome research. Mosher et al. (2014) reported that SMRT sequencing technology analyzed the original SMRTbell libraries for the V1-V9 amplicons (full-length 16S gene) of samples with the PacBio RS II pltform using P4/C2 sequencing chemistry. This technology can improve the single-molecule coverage during the sequencing process, enabling the identification of microorganisms in the environment at the species level, thus more comprehensively reflecting the microbial community structure of the sample, and providing information about the functional range within the bacterial genus, which is helpful to deepen understanding of the ecology and function of microorganisms (Mosher et al., 2014). In recent years, SMRT sequencing technology has been widely used in the study of microbial diversity in ensiling (Li et al., 2021, Yan et al., 2019). With the continuous development of sequencing technology, attention has been paid not only to the microbial community structure of silage, but also to functional analysis of the microbial population. Bai et al. (2021) and Xu et al. (2021) used the KEGG database and the PICRUST method in 16S rRNA amplicon sequencing to perform functional prediction of microbial community to better understand the functions of microbial community during fermentation. However, functional prediction methods cannot fully describe the genetic map of microbial populations. Silage microorganisms produce large amounts of metabolites during the fermentation process, which are essential for improving the fermentation quality and flavor, and for maintaining aerobic stability of silage. Metabolomics techniques can be used to qualitatively analyze the functions of microbial populations by identifying accurate metabolites during silage fermentation. Guan et al. (2020) used microbiome and metabolomic techniques to analyze the microbial population and metabolites of Napier grass during silage fermentation and aerobic exposure, and found that with a decrease in *Weissella* spp. and an increase in *Acetobacter* spp., the related metabolites were mainly related to odor, while more antimicrobial activity metabolites were found in the silage of the strain-treated group. Xu et al. (2021) used a multi-omics approach to study the regulation and interaction of bacterial microbiota and metabolome in whole crop corn ensiling systems, and found that the metabolites with biofunctions were widely correlated with the main types of lactic acid bacteria in silage, which affected the fermentation process of corn ensiling systems.

To the best of our knowledge, few studies have used microbiome and metabolomic techniques to analyze the effects of different growth stages on ryegrass silage fermentation quality, microbial community characteristics, and fermentation mechanisms. We hypothesized that growth stage affects the fermentation mechanisms of ensiling. Therefore, this study aimed to investigate the effects of different growth stages on the fermentation quality, microbial community, metabolic pathways and metabolomic characteristics of ryegrass silage by combining SMRT sequencing and metabolomics technology, so as to reveal the interaction mechanism between ryegrass raw materials at different growth stages and the attached microorganisms in the fermentation process of ensiling. This study aimed to further understand the relationship between the main fermentation microorganisms and metabolites, in order to provide new ideas for screening functional lactic acid bacteria and constructing an evaluation framework of silage with biological functions, thus laying a theoretical foundation for the production of safe and high-quality silage for animal consumption.

## Materials and methods

### Raw materials and silage preparation

Italian ryegrass was planted on June 19, 2021, at an experiment field in Siziwangqi Banner (111°33′E, 41°32′N, Inner Mongolia, China). It was harvested at booting stage on August 7, 2021, initial flowering stage on August 17, 2021, and full flowering stage on August 27, 2021 (June–August: monthly mean temperatures 18.7, 20.8, and 17.0°C; monthly total precipitation: 23.8, 41.2, and 45.8 mm). The fresh materials were harvested from three randomly selected locations within the field as three repetitions using a forage cutter (Mode-8,200; Minghong Business, Shandong, China) and chopped into 2 cm segments and left to sun-dry. Sun-drying continued until dry matter content was about 35%. From each repetition, 300 g samples were collected in sterilized bags and placed in an ice box, and immediately sent to the laboratory for determination of microbial community and chemical characteristics (Table 1). The rest of the prepared ryegrass (500 g) was packed into polyethylene plastic bags (size: 300 mm × 400 mm; Embossed Food Saver Bag Co., Ltd., Chengdu, China) and vacuum sealed with a vacuum sealer (DZ-400, Shandong Zhucheng Yizhong Machinery Co., Ltd., Zhucheng, China). These silage samples were stored at room temperature (i.e., 24–26°C) for 60 days under sheltered conditions.

**Table 1:** Chemical and microbial compositions of fresh materials.

Items	Growth stage			SEM	*p*-value
	Booting	Initial flowering	Full flowering		
DM (%)	35.04a	35.45a	31.55b	1.95	0.015
CP (% of DM)	23.54a	23.14a	19.61b	1.90	<0.001
NDF (% of DM)	50.86	53.56	54.28	2.11	0.279
ADF (% of DM)	28.21	28.30	28.40	0.45	0.410
WSC (% of DM)	9.23	9.81	10.21	0.53	0.280
Lactic acid bacteria (log_10_ cfu/g FM)	3.67	3.76	3.81	0.28	0.634
Coliform bacteria (log_10_ cfu/g FM)	6.12	5.93	6.12	0.15	0.170
Yeast (log_10_ cfu/g FM)	6.08b	6.14b	6.57a	0.27	0.121
Molds (log_10_ cfu/g FM)	3.52c	4.32a	4.16b	0.37	<0.001
pH	6.71a	6.60ab	6.44b	0.14	0.068

FM, fresh matter; DM, dry matter; CP, crude protein; NDF, neutral detergent fiber; ADF, acid detergent fiber; WSC, water-soluble carbohydrate; SEM, standard error of the mean; cfu, colony-forming unit; Means with different letters in the same row (a–c) differ significantly from each other (*p* < 0.05).

### Analysis of chemical profile and fermentation products

Measurement of each key variable (i.e., chemical composition, fermentation characteristics, and microbial counts) of each sample of raw materials and silage was repeated with three replicates. The determination of metabolomics was performed with six replicates. After ensiling, each sample was divided into two equal parts (three replicates × 2 = six replicates). From each sample, 20 g was taken and placed in a sterilized cryopreservation tube. The cryopreservation tube was immediately placed in liquid nitrogen for quick freezing for 15 min, and then stored at −80°C. These samples (three treatments × 6 replicates = 27) were sent to Majorbio Bio-Pharm Technology (Majorbio Bio-Pharm Technology Co. Ltd., Shanghai, China) for metabolomics determination.

The dry matter (DM) content of raw materials and ryegrass silages were determined by drying in a forced air oven at 65°C for 48 h and then ground to pass a 1-mm screen (FM100, Taisite Instrument Co. Ltd., Tianjin, China) for chemical analysis. Total nitrogen (TN) concentrations were determined with a Kjeldahl 8,400 autoanalyzer (Foss Co. Ltd., Hillerød, Denmark) according to the Kjeldahl method, and crude protein (CP) content was calculated as TN × 6.25. Neutral detergent fiber (NDF) and acid detergent fiber (ADF) contents were analyzed using an Ankom A2000i fiber analyzer (A2000i, Ankom Technology, Macedon, NY, United States) following the Van Soest method (Van Soest et al., 1991). Water-soluble carbohydrate (WSC) content was analyzed by anthrone reagent colorimetry (Murphy, 1958).

In order to analyze the fermentation characteristics of forage, 10 g samples of silage were combined with 90 g of deionized water and kept in a 4°C fridge for 24 h (Lu et al., 2021). The liquid extract was filtered using four layers of gauze and filter paper and used for the following analysis. The pH of the sample was measured with a glass electrode pH meter (pH S-3C, LEICI, Shanghai, China). The organic acid, including lactic acid (LA), acetic acid (AA), propionic acid (PA), and butyric acid (BA) content of silage was determined using high performance liquid chromatography (Model: e2695, Waters Corpration, Massachusetts, United States) as described by Cheng et al. (2021). The ammonia nitrogen (AN) was determined by the phenol-hypochlorite reaction (Broderick and Kang, 1980).

The plate count method described by Lu et al. (2021) was used to measure the numbers of LAB, coliform bacteria, yeast, and molds of the materials. Ten gram of fresh material or silage samples were shaken with 90 ml of sterile sterilized water at 120 rpm for 2 h, and the extracts were serially diluted for microbial counting. The colonies of lactic acid bacteria (LAB) were anaerobically cultured on De Man Rogosa Sharpe agar medium at 37°C for 48 h, and the colonies were counted. Coliform bacteria were aerobically incubated at 37°C for 48 h on violet-red bile agar culture media. Yeasts and molds were aerobically incubated at 30°C for 48 h on potato dextrose agar culture media. All culture media were from the same manufacturer (Guangzhou Huankai Microbial Science and Technology Co. Ltd., Guangzhou, China).

### Microbial diversity analysis

The Fast DNA SPIN for Soil kit (MP Biomedicals, Solon, United States) was used to extract genomic DNA from the microbial communities of the fresh and silage ryegrass samples. The concentration and purity of the DNA were evaluated using a Nanodrop 2000 UV–Vis spectrophotometer (Thermo Scientific, Wilmington, United States). The primer pairs 27F and 1492R across the full-length bacterial 16S rRNA gene were amplified using an ABI GeneAmp® 9,700 PCR thermocycler (ABI, CA, United States).

The PCR amplification procedure was performed following the methods of Li et al. (2022c). The resulting PCR product was extracted from a 2% agarose gel and further purified using AMPure® PB beads (Pacifc Biosciences, CA, United States). The purified product was mixed in equal molars and a DNA library was constructed using SMRTbell® Express Template Prep Kit 2.0 (Pacifc Biosciences, CA, United States) according to the manufacturer’s instructions. The purified SMRTbell library was sequenced using single-molecule real-time (SMRT) sequencing technology on the Pacbio Sequel II System (Pacifc Biosciences, CA, United States). The original 16S rRNA gene sequencing data were spliced using FLASH (Magoc and Salzberg, 2011). UPARSE was used to cluster operational taxonomic units (OTUs) with a similarity cutoff of 97%. Each OTU representative sequence was analyzed using the RDP Classifier (Version 2.2; Wang et al., 2007) classifier algorithm and database (nt_v20210917), and the confidence threshold was 70%.

Bioinformatic analysis of the silage microbial diversity was performed on the Majorbio Cloud platform.^1^ Following the methods of Schloss et al. (2009), alpha diversity indices calculated using Mothur (Version 1.30.1) based on OTUs information included observed OTUs, Chao1 richness, Shannon index, and Good’s coverage. Following OTU clustering, Venn diagrams were drawn using the Venn diagram package (Version 1.2) in R statistical tools (Du et al., 2021a). The similarity between microbial communities in different samples was determined by principal coordinate analysis (PCoA) based on Bray–Curtis dissimilarity using the Vegan (Version 2.5-3) package. Hierarchical cluster analysis and similarity analysis (ANOSIM) heat maps were plotted using R software (Version 3.0.2; Du et al., 2021a).

### Metabolites analysis

As described by Zhang et al. (2019), 50 mg of solid silage ryegrass samples were put in an EP tube and the metabolites were extracted with 400 μl methanol: water (4: 1, v / v) solution. According to the method described by Fu et al. (2022), a mixed quality control sample (QC) was prepared. The processing and testing of QC samples were the same as those of analytical samples. The instrument platform of this LC–MS study used a Thermo Fisher ultra-high performance liquid chromatography tandem Fourier transform mass spectrometry UHPLC-quadrupole-electrostatic field orbital trap high resolution mass spectrometer HF-X system. UHPLC–MS/MS analysis was performed according to the method described by Yang et al. (2022).

Multivariate statistical analysis was performed using the ropls (Version1.6.2) R software package, including principal component analysis (PCA), partial least-square discriminant analysis (PLSDA) and orthogonal projections to latent structure discriminant analysis (OPLS-DA). The differential metabolites were detected based on OPLS-DA analysis to obtain the first principal component (VIP) of variable importance in the projection. Combined with Student t test, the screening conditions for the main differential metabolites were: *p* < 0.05 and VIP>1. The variables of all metabolites were scaled to the comfort variance, and the unsupervised principal component analysis (PCA) method was used to describe the overall clustering, trends, and outliers to obtain an overview of the metabolic data. Metabolite sets were formed by screening statistically significant (*p* < 0.05) differential metabolites. Single factor analysis of variance (ANOVA) was used to compare the silage samples at three different growth stages. A total of 442 differential metabolites were summarized as the metabolite set in this analysis. Metabolite enrichment analysis and metabolic pathway analysis were performed using database searches to establish links to biochemical pathways. The functional characteristics and classification of differential metabolites were analyzed using the Kyoto Encyclopedia of Genes and Genomes (KEGG) database (Tian et al., 2022). These metabolites can be classified according to the pathway they take or the function they perform. Enrichment analysis is often used to identify whether a group of metabolites appears at a functional node. Fisher’s exact test was used to find statistically significant enrichment pathways.

### Statistical analysis

The variance analysis (ANOVA) of fermentation, nutritional characteristics and microbial populations of fresh materials and silage samples were performed using the general linear model program (GLM) to test the differences between samples in SAS software (ver. 9.3; SAS Institute Inc., Cary, NC, United States). One-way analysis of variance (ANOVA) and Duncan’s multiple range test were used to evaluate differences between treatments. When *p* < 0.05, the effect was considered significant. Microbiota and metabolome data were processed using the Majorbio I-Sanger Cloud Platform^2^ online platform.

## Results

### Chemical composition and microbial populations of raw materials

The chemical composition and microbial populations of raw materials at different growth stages are shown in Table 1. Dry matter (DM) content varied from 31.55 to 35.45%. The CP content at the booting stage was significantly higher than at the full flowering stage (*p* < 0.05) and CP decreased at later growth periods. The contents of ADF and NDF and the amount of LAB tended to increase at later growth stages, but the differences were not significant. There was no significant different in WSC, although WSC content increased at later growth periods and the WSC content of all raw materials was higher than 9% DM. There were no significant differences in LAB or coliform bacteria populations among the growth stages, but the yeast and mold populations of the booting stage sample was significantly lower than at later stages (*p* < 0.05).

### Silage quality of ryegrass silage at different growth stages

The fermentation quality of ryegrass silages made with materials at different growth stages is shown in Table 2. After ensiling, there were significant effects of growth stage on pH values, AN/TN, LA, AA, DM and WSC content (*p* < 0.05). The pH values of ryegrass silages at different growth stages ranged from 4.67 to 6.18. Booting stage silage achieved significantly lower pH (4.67) compared to other stages (*p* < 0.05), and its LA was significantly higher (74.72 g/kg) than other stages (*p* < 0.05). Compared with the fresh materials, the DM and WSC contents in silages were lower. After fermentation, the WSC content of silages at booting stage decreased the most, and its WSC content was the lowest and significantly lower than silages made with materials at the initial flowering stage (*p* < 0.05). The WSC content (8.22% DM) and pH value (6.18) of silage at the initial flowering stage were significantly higher than at other stages (*p* < 0.05), and its LA content was significantly lower (*p* < 0.05). These results indicated that silage made with material harvested at the initial flowering stage was not well fermented.

**Table 2:** Silage quality of ryegrass silage at different growth stages.

Items	Growth stage			SEM	*p*-value
	Booting	Initial flowering	Full flowering		
pH	4.67c	6.18a	4.86b	0.72	<0.001
AN/TN, %	1.15c	3.31b	5.26a	1.92	0.003
Lactic acid, g/kg DM	74.72a	27.52c	58.42b	21.61	0.003
Acetic acid, g/kg DM	7.77c	12.32b	23.70a	7.26	0.002
Propionic acid, g/kg DM	13.05	10.26	11.50	1.55	0.339
Butyric acid, g/kg DM	6.47	6.58	6.49	0.83	0.906
DM (%)	34.43b	35.23a	30.86b	2.07	<0.001
WSC (% of DM)	5.12b	8.22a	5.46b	1.49	<0.001

FM, fresh matter; DM, dry matter; AN/TN, ammonia nitrogen/total nitrogen; WSC, water-soluble carbohydrate; SEM, standard error of the mean; Means with different letters in the same row (a–c) differ significantly from each other (*p* < 0.05).

### The microbial community and species diversity of fresh materials and silage samples

**Figure 1:**
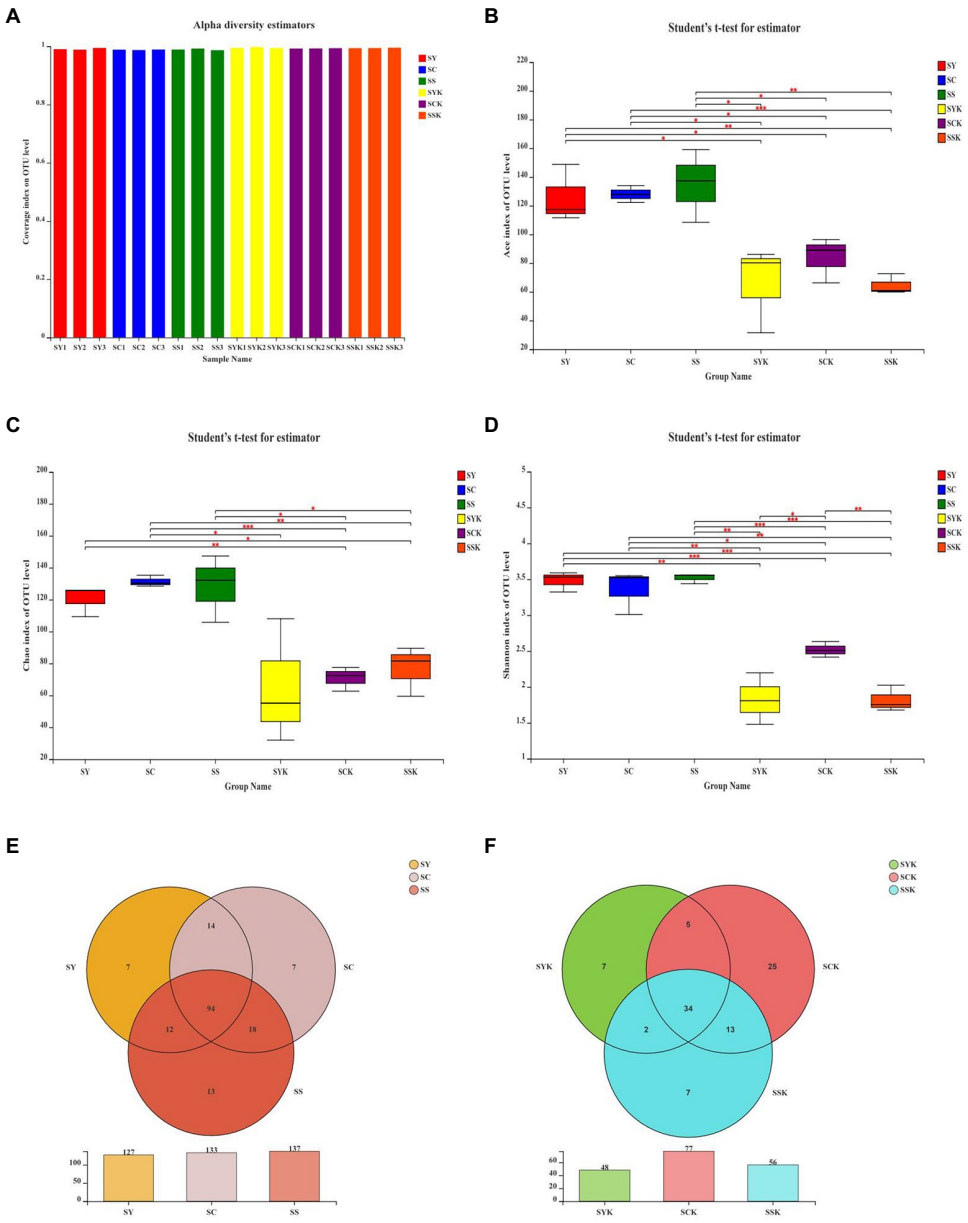
Alpha-diversity of the bacterial community of fresh materials and ryegrass silages and Venn analysis of OTUs. **(A–D)** the Coverage, ACE, Chao1 and Shannon index of the bacterial community of fresh materials and ryegrass silages, respectively. ‘*,’ ‘**,’ and ‘***’ represent *p* < 0.05, *p* < 0.01, and *p* < 0.001, respectively. **(E)** Venn analysis of OTUs in the bacterial community of fresh materials; **(F)** Venn analysis of OTUs in the bacterial community of ryegrass silage; SY, Fresh materials at booting stage; SC, Fresh materials at initial flowering; SS, Fresh materials at full flowering; SYK, Silage samples at booting stage; SCK, Silage samples at initial flowering; SSK, Silage samples at full flowering.

As shown in Figure 1, we performed SMRT sequencing of the full-length 16S rRNA gene of fresh materials and silage samples, and performed Alpha diversity analysis and OTU Venn analysis of bacterial communities. Based on the coverage values for all samples (Figure 1A), coverage greater than 99% fully captures most bacterial communities. Compared with fresh materials, the Ace (Figure 1B), Chao 1 (Figure 1C) and Shannon indices (Figure 1D) in each treated silage decreased. At the same time, there were significant differences among the treatment groups, indicating that growth stage had significant effects on the microbial diversity of ryegrass silage. The OTUs showed similar trends to those of the Ace, Chao1 and Shannon indices. Compared to fresh materials, the number of OTUs decreased in all treatment groups after ensiling. The core microbiomes of fresh materials (Figure 1E) were composed of 94 shared OTUs and 7,7 and 13 unique OTUs, respectively. After ensiling, the number of core microorganisms in each treatment group was 34, and the number of unique microorganisms was 7, 25, and 7, respectively (Figure 1F).

### Bacterial community compositions of fresh materials and silage samples

**Figure 2:**
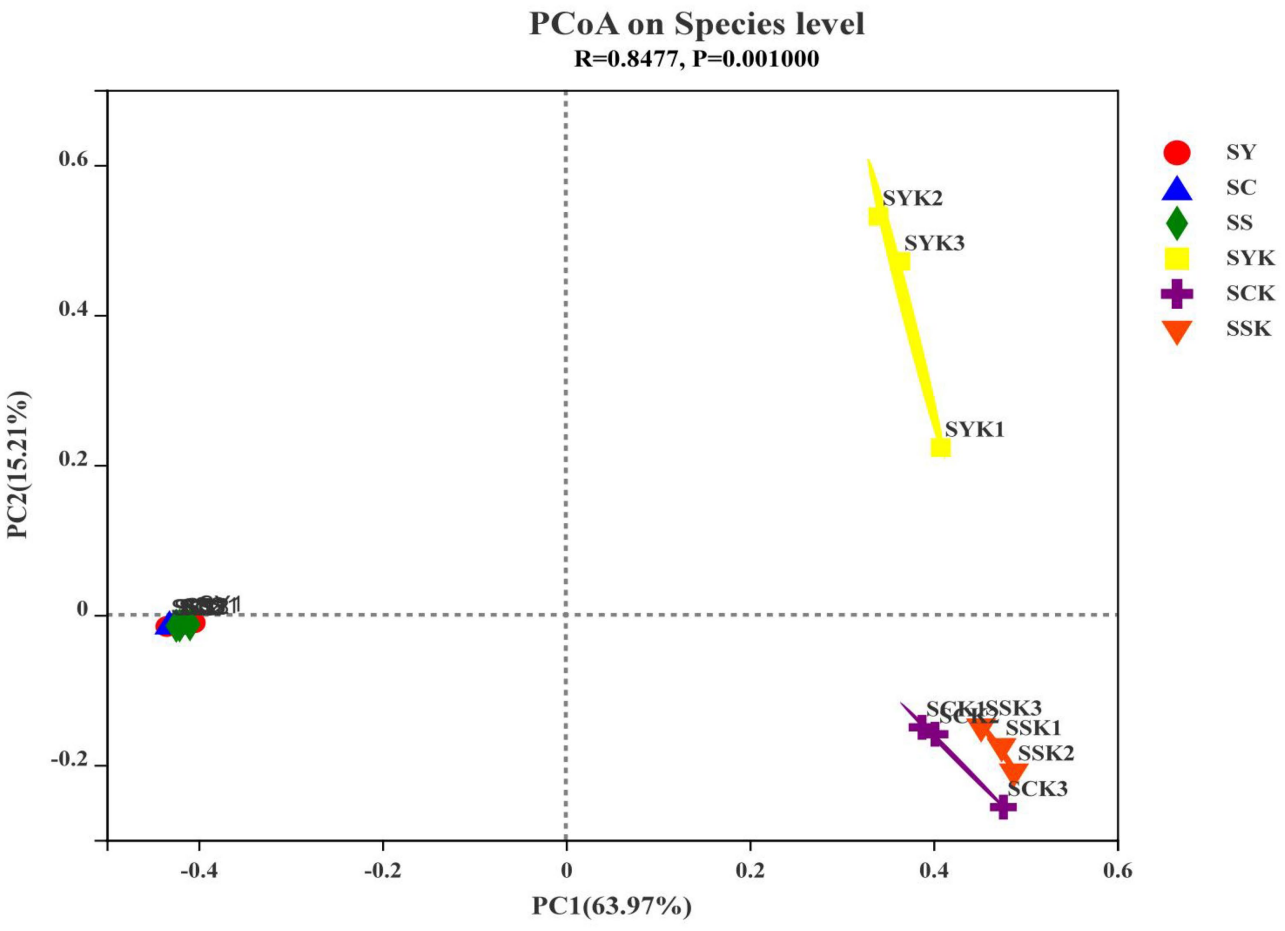
The principal coordinate analysis (PCoA) plot showing variation in bacterial community structure of growth stages. Each point represents an individual. SY, Fresh materials at booting stage; SC, Fresh materials at initial flowering; SS, Fresh materials at full flowering; SYK, Silage samples at booting stage; SCK, Silage samples at initial flowering; SSK, Silage samples at full flowering.

We confirmed bacterial community differences between fresh material and ryegrass silage using β-diversity analysis through principal coordinates analysis (PCoA; Figure 2). Using the Bray-Curtis distance algorithm and the between-group difference test at the OTU classification level, we observed no significant separation between bacterial communities of fresh materials, but significant separation after ensiling.

**Figure 3:**
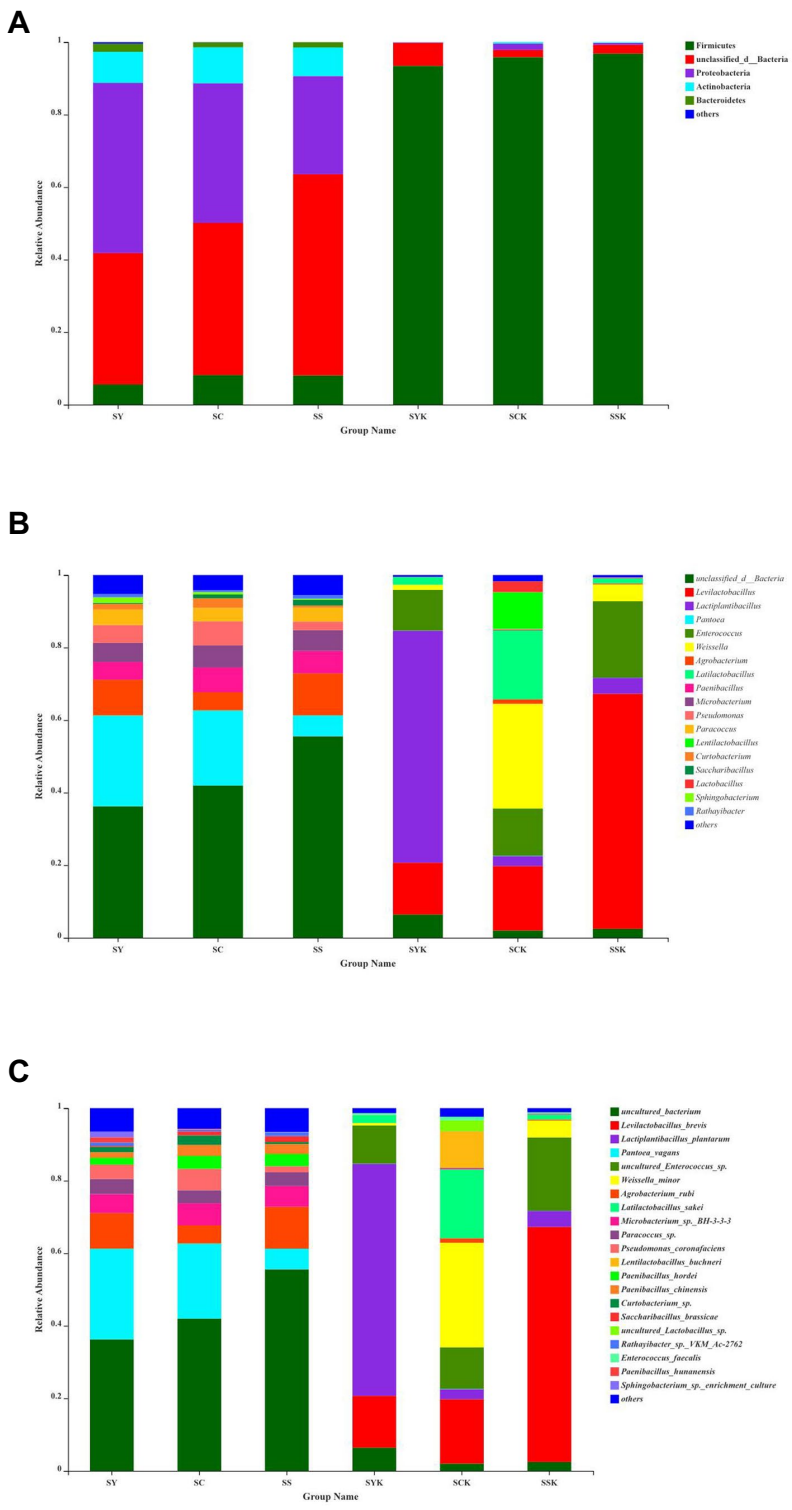
Bacterial community composition at phylum **(A)**, genus **(B)**, and species **(C)** level in fresh materials and ryegrass silage by SMRT. SY, Fresh materials at booting stage; SC, Fresh materials at initial flowering; SS, Fresh materials at full flowering; SYK, Silage samples at booting stage; SCK, Silage samples at initial flowering; SSK, Silage samples at full flowering.

The relative abundances of the bacterial community composition at the phylum (Figure 3A), genus (Figure 3B), and species (Figure 3C) level in fresh materials and silage samples by SMRT sequencing are shown in Figure 3. Proteobacteria and unclassified bacteria were the main phyla in fresh materials. After 60 d of fermentation, Proteobacteria abundance decreased significantly, while Firmicutes abundance increased, becoming the most dominant bacteria at the phylum level. At the genus level, the relative abundance of unclassified bacteria was higher in ryegrass raw materials (SY, SC and SS). However, after fermentation, the relative abundance of *Lactiplantibacillus* (63.98%) was the highest in the SYK group; In the SCK group, the relative abundance of *Weissella* (28.85%), *Latilactobacillus* (18.97%), *Levilactobacillus* (17.77%), *Enterococcus* (13.03%), and *Lentilactobacillus* (10.09%) were the highest, and in the SSK group, the relative abundance of *Levilactobacillus* (64.81%) was the highest. At the species level, the dominant species in ryegrass raw materials (SY, SC and SS) were uncultured bacterium, *Pantoea vagans* and *Agrobacterium rubi*. After ensiling, *Lactiplantibacillus plantarum* (63.98%) was the dominant species in SYK. *Weissella minor* (28.82%), *Latilactobacillus sakei* (18.97%), *Levilactobacillus brevis* (17.77%), *Enterococcus* sp. (11.45%), and *Lentilactobacillus buchneri* (10.09%) were the dominant species in SCK, and *Levilactobacillus brevis* (64.81%) was the dominant species in SSK.

### Correlations between the microbial community and fermentation products

**Figure 4:**
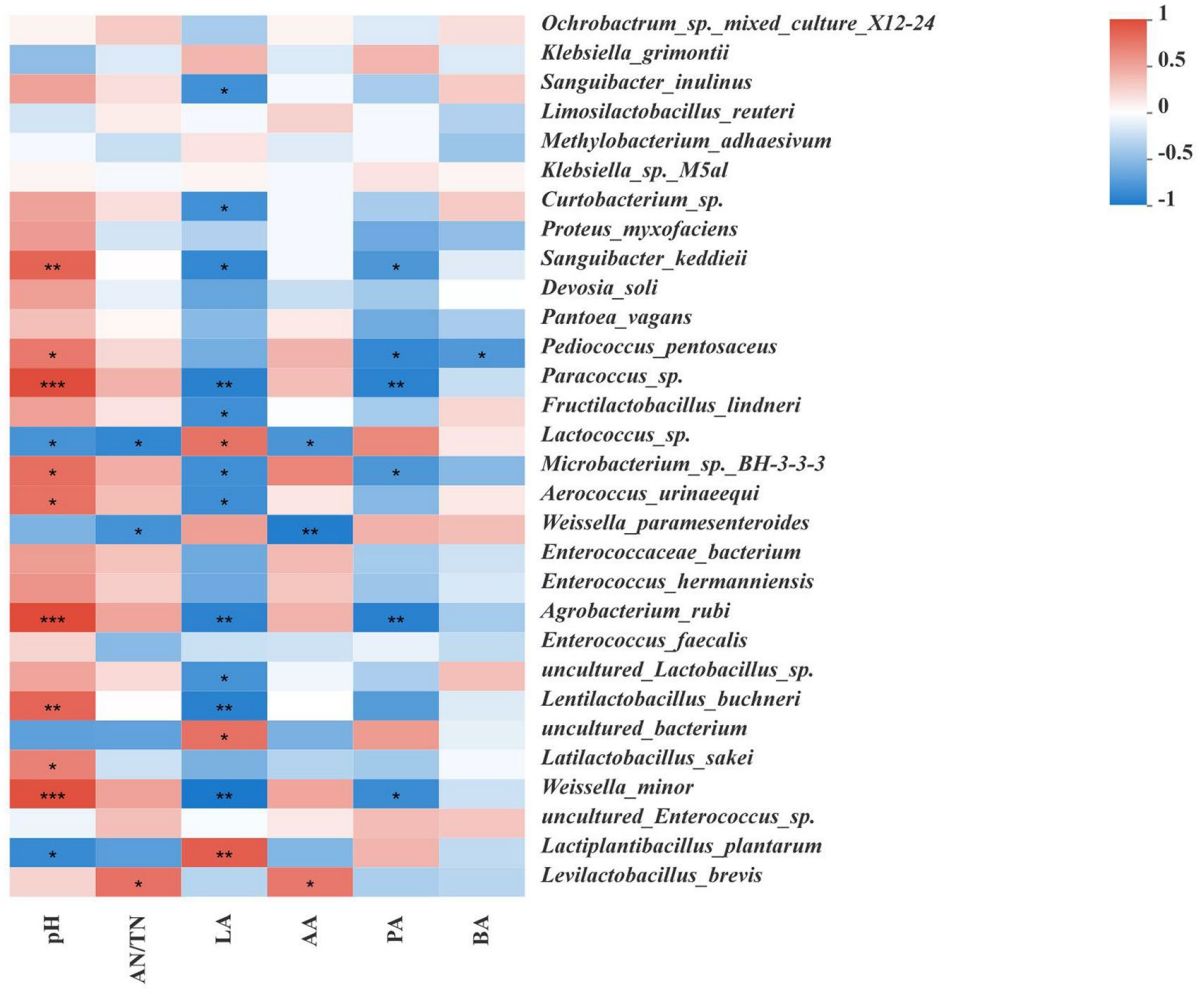
Correlation analysis of the bacterial communities with fermentation characteristics. Fermentation characteristics are displayed vertically and the bacterial community information is displayed horizontally. The corresponding values displayed in the heat map are the Spearman correlation coefficients r, which range between −1 and 1; *r* < 0 indicates a negative correlation (blue), *r* > 0 indicates a positive correction (red), and ‘*,’ ‘**,’ and ‘***’ represent *p* < 0.05, *p* < 0.01, and *p* < 0.001, respectively.

Figure 4 shows Spearman correlations between the microbial community and fermentation products at the species level. pH was positively correlated with the presence of *Paracoccus* sp., *Lentilactobacillus buchneri*, *Latilactobacillus sakei* and *Weissella minor*, and negatively correlated with the presence of *Lactiplantibacillus plantarum* and *Lactococcus* sp. AN/TN level was positively correlated with the presence of *Levilactobacillus brevis*, while negatively correlated with the presence of *Weissella paramesenteroides* and *Lactococcus* sp. LA level was positively correlated with the presence of *Lactococcus* sp. and *Lactiplantibacillus plantarum*, and negatively correlated with the presence of *Paracoccus* sp., *Lentilactobacillus buchneri* and *Weissella minor*. AA level was positively correlated with the presence of *Levilactobacillus brevis*, and negatively correlated with the presence of *Weissella paramesenteroides* and *Lactococcus* sp. PA level was negatively correlated with the presence of *Pediococcus pentosaceus* and *Weissella minor*. BA level was negatively correlated with the presence of *Pediococcus pentosaceus*.

### Identification and metabolic characterization of ryegrass silage metabolites

The main differential metabolites of ryegrass silage at different growth stages are shown in Table 3, and all the differential metabolites in the table were significantly different. In SYK group, D-mannitol, acetylcholine, L-asparagine, guanine, cytosine, N-formylmethionine and D-Ala-D-Ala were increased, and L-arginine, citric acid, malic acid and deoxyguanosine were decreased. These metabolites showed the opposite trend in the SCK group. It was worth noting that thymine, uridine and L-glutamine appeared in the SCK group. In SSK group, D-mannitol, acetylcholine and thymine were increased, and L-asparagine, L-arginine, citric acid, malic acid, deoxyguanosine, uridine and L-glutamine were decreased.

**Table 3:** The main differential metabolites of ryegrass silage at different growth stages.

Metabolites	Relative concentration			Fold changes		
	SYK	SCK	SSK	Log2 (SYK/SCK)	Log2 (SCK/SYK)	Log2 (SSK/SCK)
D-Mannitol	7.43	7.04	7.90	12.92	−12.92	6.01
Acetylcholine	6.00	4.34	6.30	2.14	−2.14	1.86
L-Asparagine	5.84	5.23	4.71	6.29	−6.29	−6.61
L-Arginine	5.17	6.01	4.54	−4.64	4.64	−2.47
Citric acid	6.29	6.71	6.19	−10.74	10.75	−8.67
Malic acid	7.07	8.25	7.14	−4.50	4.50	−4.79
Deoxyguanosine	6.39	6.90	5.67	−9.09	9.09	−3.53
Guanine	7.23	6.76	-	10.22	−10.22	-
Cytosine	5.35	4.89	-	7.68	−7.68	-
N-Formylmethionine	6.01	5.59	-	9.57	−9.57	-
D-Ala-D-Ala	5.12	4.70	-	8.04	−8.04	-
Thymine	-	5.91	6.23	-	-	12.95
Uridine	-	7.36	6.92	-	-	−11.20
L-Glutamine	-	5.42	4.17	-	-	−2.66

The relative concentrations of metabolites were derived from the average data of 6 biologically repeated samples of LC–MS. Use the formula Log2 (X/Y) to calculate the fold change. X and Y represent different growth stages: SYK, Silage samples at booting stage; SCK, Silage samples at initial flowering; SSK, Silage samples at full flowering.

**Figure 5:**
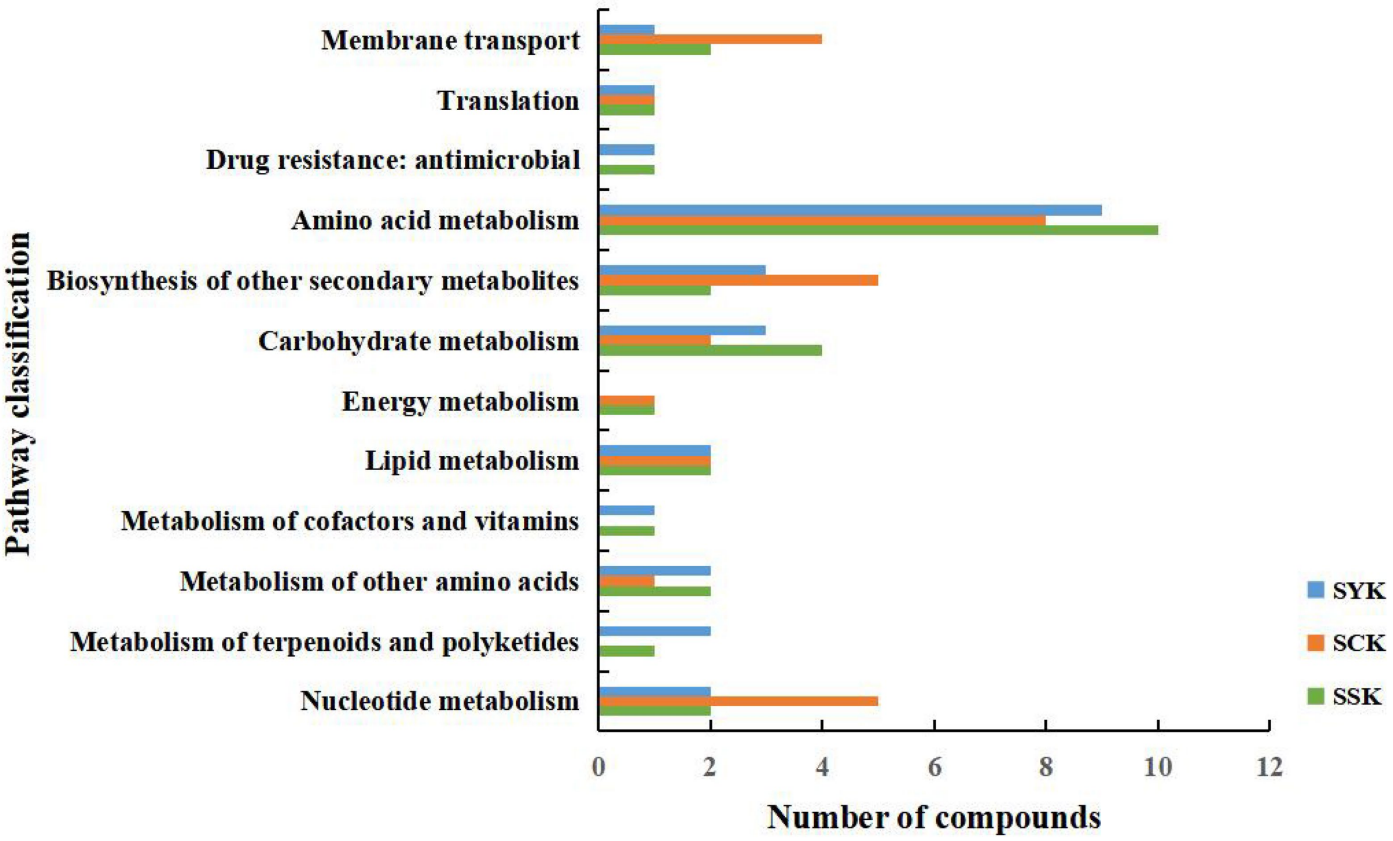
Comparison of KEGG functional pathways of main differential metabolites of ryegrass silage at different growth stages in positive and negative ion modes. SYK, Silage samples at booting stage; SCK, Silage samples at initial flowering; SSK, Silage samples at full flowering.

As shown in Figure 5, we compared the KEGG functional pathways of the main differential metabolites of ryegrass silage at different growth stages under positive and negative ion modes. According to the KEGG pathway database classification, the KEGG functional pathways of metabolites after ensiling at different growth stages mainly involved four pathways: metabolism, human diseases, genetic information processing and environmental information processing, but mainly focused on metabolism. The vertical axis of Figure 5 is the secondary classification of the KEGG metabolic pathway, and the horizontal axis is the number of metabolites in this pathway. In booting stage (SYK), the amino acid metabolism pathway had the largest number of annotated metabolites, with 9 metabolites annotated, followed by the biosynthesis of other secondary metabolites and carbohydrate metabolism, with 3 metabolites annotated, respectively. In initial flowering (SCK), the amino acid metabolism pathway had the largest number of annotated metabolites, with 8 metabolites annotated, followed by the biosynthesis of other secondary metabolites and nucleotide metabolism, with 5 metabolites annotated, respectively. In full flowering (SSK), the amino acid metabolism pathway had the largest number of annotated metabolites, with 10 metabolites annotated, followed by the carbohydrate metabolism, with four metabolites annotated.

**Figure 6:**
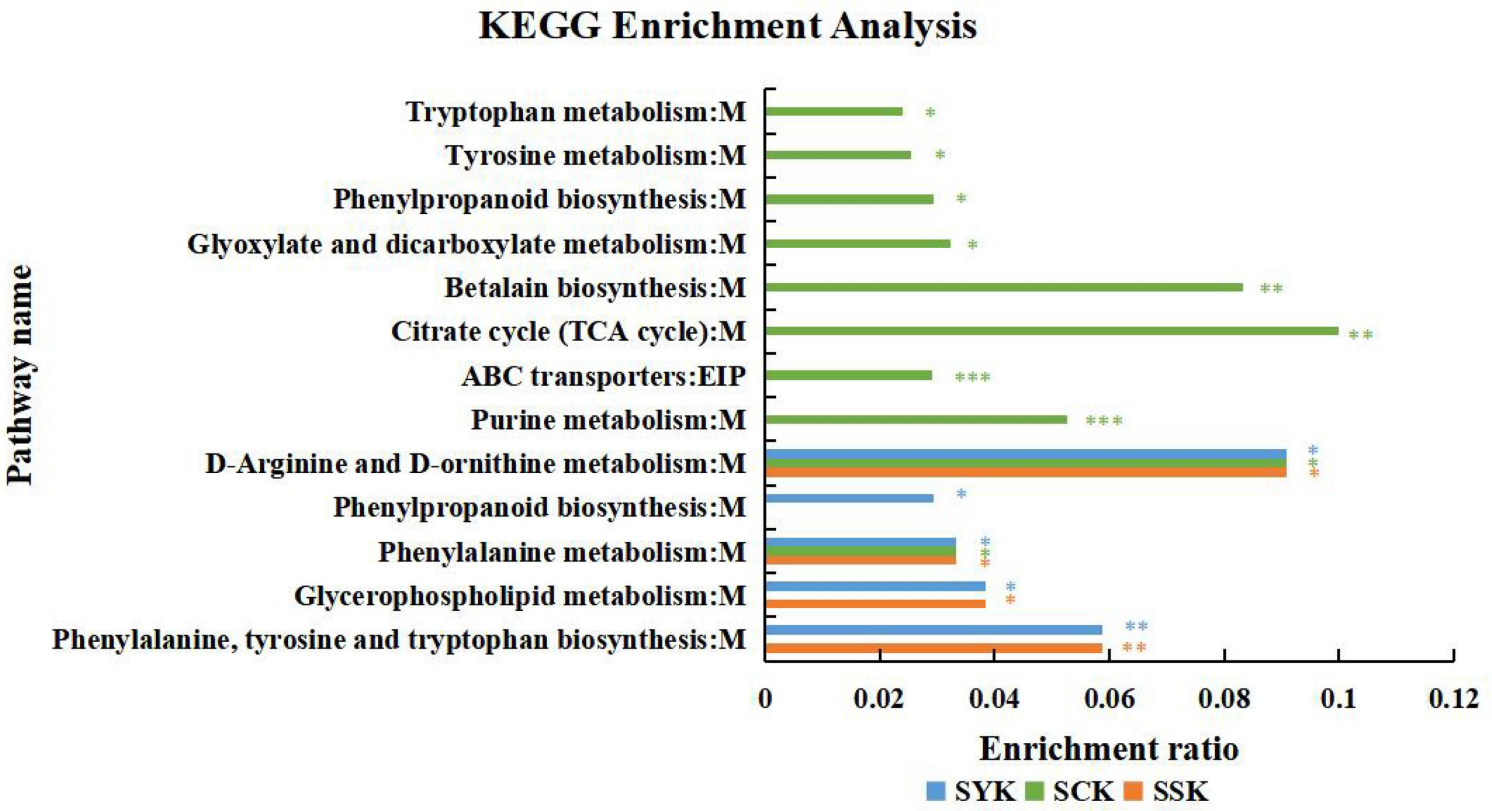
KEGG pathway enrichment analysis of the main differential metabolites of ryegrass silage at different growth stages in positive and negative ion modes (SYK, Booting stage; SCK, Initial flowering; SSK, Full flowering). M, and EIP are the class names of metabolic pathways in KEGG annotation. M: Metabolism; EIP: Environmental Information Processing. *value of p*-uncorrected <0.05 and column chart showing *value of p* values for the top 20 pathways; *p < 0.05; **p < 0.01, ***p < 0.001.

In addition, Figure 6 shows the results of the KEGG pathway enrichment analysis of the main differential metabolites of ryegrass silage at different growth stages under positive and negative ion modes. The ordinate in the figure represents the pathway name, and the abscissa represents the enrichment rate, which indicates the ratio of the numbers of enriched metabolites in the pathway to the background number annotated to the pathway. The larger the value, the greater the degree of enrichment. In booting stage (SYK), there were 5 significantly enriched pathways, among which phenylalanine, tyrosine and tryptophan biosynthesis were significantly enriched (*p* < 0.01), and D-arginine and D-ornithine metabolism had the highest enrichment rate (0.0909). In initial flowering (SCK), there were 10 significantly enriched pathways, among which purine metabolism and ABC transporters were significantly enriched (*p* < 0.001), and citrate cycle (TCA cycle) had the highest enrichment rate (0.1000). In full flowering (SSK), there were four significantly enriched pathways, among which phenylalanine, tyrosine and tryptophan biosynthesis were significantly enriched (*p* < 0.01), and D-arginine and D-ornithine metabolism had the highest enrichment rate (0.0909). This was similar to the results of the booting stage, but lacks phenylpropanoid biosynthesis compared to booting stage. According to the classification of KEGG pathway database, except for ABC transporters, which are related to environmental information processing, the remaining metabolites belong to the metabolic class.

### Correlations between the relative abundances of bacteria and metabolites

**Figure 7:**
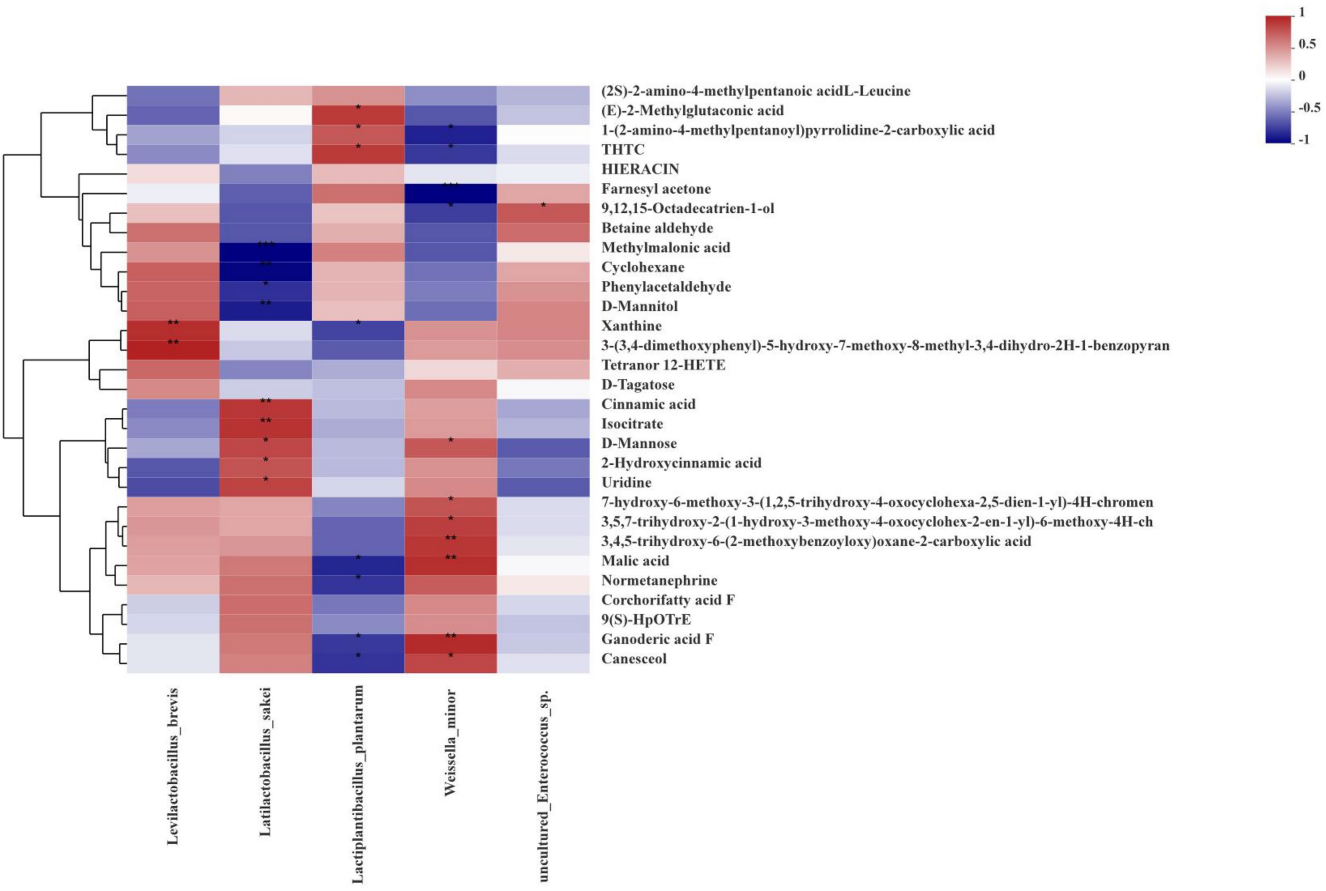
Correlation analysis of high abundance species-level bacteria and metabolites in silage at different growth stages. The corresponding values in the heat map are the Spearman correlation coefficients, r, which range between −1 and 1; *r* < 0 indicates a negative correlation (blue), *r* > 0 indicates a positive correction (red), and ‘*’, ‘**’ and ‘***’ represent *p* < 0.05, *p* < 0.01, and *p* < 0.001, respectively.

Spearman correlations between the fermentation bacterial community and differential metabolites at the level of species in ryegrass silages at days 60 are described in Figure 7, along with those bacteria in silage at different growth stages that were co-occurring or highly enriched. After fermentation, the abundance of *Levilactobacillus brevis* had a positive correlation with xanthine and 3-(3,4-dimethoxyphenyl)-5-hydroxy-7-methoxy-8-methyl-3,4-dihydro-2H-1-benzopyran. The abundance of *Latilactobacillus sakei* had positive correlations with cinnamic acid, isocitrate, D-Mannose, 2-Hydroxycinnamic acid and uridine, but had negative correlations with methylmalonic acid, cyclohexane, phenylacetaldehyde and D-Mannitol. The abundance of *Lactiplantibacillus plantarum* was positively correlated with (E)-2-Methylglutaconic acid, 1-(2-amino-4-methylpentanoyl)pyrrolidine-2-carboxylic acid and THTC, but negatively correlated with xanthine, malic acid, ganoderic acid F and canesceol. The abundance of *Weissella minor* had a positive correlation with D-Mannose, malic acid, ganoderic acid F and canesceol, but a negative correlation with 1-(2-amino-4-methylpentanoyl)pyrrolidine-2-carboxylic acid, THTC and farnesyl acetone.

## Discussion

### Chemical composition and microbial population of raw materials

Ensiling is an anaerobic fermentation process in which microorganisms attached to the surface of raw materials interact with chemical components (Wang et al., 2022a). The composition of attached microbial communities and chemical components of raw materials play important roles in determining the fermentation quality of silage (Wang et al., 2022a). An adequate number of epiphytic LAB (5.0 log_10_ CFU per gram FM) and WSC content (5% DM) of raw materials are considered key factors to ensure acceptable fermentation quality (Long et al., 2022; Wang et al., 2017). In the present study, the WSC content (>9% DM) of ryegrass raw materials at different growth stages could provide sufficient fermentation substrate for extensive fermentation. However, the number of LAB (3.67–3.81 log_10_ CFU per gram FM) was lower than the theoretical requirement to ensure good fermentation, which may cause poor fermentation quality. In addition, the number of undesirable microorganisms (coliform bacteria, yeast and molds) in ryegrass raw materials at different growth stages was high, which could compete with LAB for limited WSC content and may be detrimental to fermentation (Wang et al., 2022a). According to Xie et al. (2012) and Lounglawan et al. (2014), the number of microorganisms on plant surfaces vary at different growth stages, and the number of epiphytic LAB increase with the delay of harvest date (Long et al., 2022). In this study, when harvested at later growth stages, the number of LAB showed an increasing trend, which was consistent with the findings of Long et al. (2022). Nazli et al. (2019) reported that the chemical composition and nutritional value of forage varied between the growth stages. With the increase of growth days, the CP contents of ryegrass decreased, and the contents of NDF and ADF increased, which was consistent with the results of Long et al. (2022) in King grass harvested at different dates. The decrease in CP content was due to the decrease in leaf to stem ration as forage grew (Contreras-Govea and Albrecht, 2006), while the increase in ADF and NDF can be attributed to the lignification process as harvest dates were delayed (Lauer and Darby, 2002).

### Silage quality of growth stages ryegrass silage

After ensiling, the dry matter and WSC contents of ryegrass silage decreased due to the metabolic activities of aerobic microorganisms during fermentation (Yuan et al., 2017). Under the action of microorganisms, WSC was consumed, utilized and decomposed into organic acids, resulting in a decrease in pH value (Tian et al., 2022). Wang et al. (2020) reported that the pH value of relatively high-quality silage should be less than 4.2, and butyric acid content should be less than 10 g/kg DM. In our study, the butyric acid content of all treatments were lower than 10 g/kg DM, while the pH values were higher than 4.2, because no exogenous additives were added. Wang et al. (2018) also found that the pH values of ryegrass after natural fermentation were higher than 4.2. However, the pH values of silage at booting stage (4.67) and full flowering stage (4.86) were lower than 5.0, which was acceptable. A pH value lower than 5.0 has been found to be conducive to the increase of lactic acid and limited the growth of adverse microorganisms (Keles et al., 2014). Lactic acid is considered to be the most effective acid causing a decrease in the pH of silage (Danner et al., 2003). The lactic acid content at booting stage was the highest (74.72 g/kg DM), followed by that at full flowering stage (58.42 g/kg DM). However, the pH value of silage at the initial flowering stage (6.18) was higher than 5.0, indicating that microorganisms did not fully ferment. The number of LAB involved in the fermentation process was low, and the content of lactic acid was also the lowest (27.52 g/kg DM) among the three treatment groups, which was also indicated by the high WSC content (8.22% DM vs. 5.12% DM and 5.46% DM) after ensiling. Ammonia nitrogen content reflects the degree of protein degradation, which is usually suggested to be less than 10% of total nitrogen (He et al., 2022). In the current study, the AN/TN ratios of all treatments were lower than 10%, which was acceptable. In addition, different microbial communities and metabolic processes at different growth stages lead to differences in pH and organic acid concentrations.

### Microbial community compositions of fresh materials and silage samples

The diversity, structure and function of microbial communities are important research topics in microbial ecology, and have long been a focus of research (Liu et al., 2019). Monitoring the diversity of the bacterial community is one of the important methods to study the effects of microorganisms on ensiling fermentation (Tian et al., 2022). In this study, the coverage value of all samples was more than 99%, indicating that sequencing depth was sufficient to represent microbial community composition. Alpha diversity represents the richness and diversity of species in ecosystems, and the Ace and Chao 1 indices are used to measure the richness of species, and the lower the index, the lower the richness. The Shannon index is used to measure species diversity, and the lower the index, the lower the diversity of species (Zhang et al., 2020). In the present study, Ace, Chao 1 and Shannon indexes decreased after ensiling, indicating that the richness and diversity of bacterial communities decreased after ensiling. According to Bai et al. (2020), when the abundance of dominant bacteria is high, the diversity of microbial communities decreases. Through Venn analysis, we also found that the number of core microorganisms after ensiling decreased from 94 to 34. This is because in the process of anaerobic fermentation, the metabolic activities of some adverse microorganisms in raw materials are inhibited, and they are gradually replaced by the dominant bacteria that dominate the fermentation process (Ni et al., 2017). The Ace index and Shannon index of the SCK group were higher than those of the other groups, indicating that the abundance of dominant bacteria in the SCK group was lower, which may lead to poor fermentation. The PCoA analysis of β-diversity further showed the changes in the microbial community before and after ensiling. We found that the bacterial communities of silage made from materials harvested at different growth stages were significantly isolated, indicating that growth stage affected the composition of fermented bacteria and thus affected the fermentation quality of silage (Ni et al., 2017).

Since the PacBio SMRT method can generate long sequence readings, it can accurately reveal the bacterial composition and dynamics during ensilement at the species level. Some recent studies (Ding et al., 2020; Bai et al., 2021; Du et al., 2021a; Xu et al., 2021; Du et al., 2022a) have used the PacBio SMRT method to reveal the complete bacterial community in the ensiling system to provide more valuable biological information (Guo et al., 2018). Xu et al. (2021) used SMRT sequencing to investigate the population dynamics of whole crop corn silage during ensiling at species level. Du et al. (2022b) used PacBio-SMRT sequencing to explore the microbial co-occurrence network and silage fermentation of gliricidia and leucaena prepared with Napier grass and corn stover in Southern Africa. Bai et al. (2022) used SMRT sequencing to study the effects of different lactic acid bacteria on the fermentation process of whole-plant corn silage stored at different temperatures based on bacterial community successions, interaction networks, and predicted functions. The SMRT sequencing technology used in this study can accurately evaluate the relative bacterial abundances of ryegrass raw materials at different growth stages and silage samples at phylum, genus and species levels. At phylum level, in addition to unclassified bacteria in the raw materials, the main bacteria were Proteobacteria. Proteobacteria play an important role in degradation of organic matter and acceleration of carbon and nitrogen cycles (Ma et al., 2018). After ensiling, Proteobacteria abundance decreased, while Firmicutes abundance increased and became the dominant bacteria in the whole fermentation process. Similar results were reported in barley silage (Ding et al., 2020; Liu et al., 2019). Wang et al. (2022a) reported that Firmicutes can produce a variety of enzymes (such as proteases, cellulase), and their acid hydrolytic function plays an important role in anaerobic environments. At genus level, unclassified bacteria and *Pantoea* were the dominant bacteria in all fresh raw materials. Du et al. (2022a) also found *Pantoea* on the raw materials of Napier grass, and some *Pantoea* sp. are pathogenic to vegetables. After 60 days of ensiling, the dominant bacteria were replaced by the homofermentative genus of *Lactiplantibacillus* (Ding et al., 2020) and the heterofermentative genus of *Levilactobacillus* and *Lentilactobacillus* (Xu et al., 2018), which promoted ensiling fermentation. This result is consistent with the acid production in SYK and SSK silage samples, and with previous studies (Yan et al., 2019; Ding et al., 2020) which also reported that *Lactobacillus* was the dominant bacteria in silage samples. At species level, the relative abundance of bacteria in different treatment groups was significantly different, resulting in significant differences in lactic acid and acetic acid content after fermentation. In the SYK group, the dominant species was *Lactiplantibacillus plantarum*, a homofermentative lactic acid bacterium that grows well in acidic environments and promotes lactic acid fermentation (Xu et al., 2018; Du et al., 2022a). During ensiling fermentation, it can use sugars to produce more lactic acid than other strains, thereby reducing pH and inhibiting the growth of harmful bacteria, resulting in high-quality silage (Weiss et al., 2016; Cai et al., 2020). This is also the reason why the lactic acid content of booting stage silage was significantly higher and the pH was significantly lower than other groups. Guo et al. (2018) reported that *Lactiplantibacillus plantarum* was the most abundant species in ensiled alfalfa during 30 to 90 days of natural fermentation. *Weissella minor* and *Latilactobacillus sakei* were the dominant species in the SCK group, which also had low abundances of *Lactiplantibacillus plantarum*, *Levilactobacillus brevis* and *Lentilactobacillus buchneri*. This may be due to the low ability of the dominant species to produce lactic acid, and the low abundance of *Lactiplantibacillus plantarum* with strong acid production ability leads to lower lactic acid content and higher pH value of the silage made with materials harvested at the initial flowering stage (Cai et al., 2020). *Levilactobacillus brevis* was the dominant species in SSK group. *L. brevis* is a heterofermentative strain that produces a part of the lactic acid and part of the acetic acid during the fermentation process, thereby improving aerobic stability (Gallo et al., 2022; Xu et al., 2018; Daniel et al., 2015). This is also the reason why the lactic acid content of silage at the full flowering stage was lower than that at the booting stage, while the acetic acid content was the highest. Ding et al. (2020) reported that *L. brevis* was the most abundant species in ensiled *Elymus nutans* grass during 60 days of fermentation. It has been reported that many factors affect the composition of bacterial communities on plant surfaces, including plant species, climate, and geographical location.

### Correlations between the microbial community and fermentation products

Microorganisms affect silage fermentation through a series of metabolites (Du et al., 2022b). For example, *Lactobacillus* mainly affects lactic acid production, while *Enterobacteria* can ferment lactic acid to produce acetic acid and other products (Guan et al., 2018). As shown in Figure 4, we mainly analyzed the correlations between dominant bacteria and fermentation characteristics in each treatment group after ensiling fermentation. The correlations between pH, lactic acid or acetic acid and bacteria in all silage samples showed that *Lactiplantibacillus plantarum*, *Lentilactobacillus buchneri*, *Levilactobacillus brevis*, *Latilactobacillus sakei*, *Weissella minor*, and *Weissella paramesenteroides* played important roles in the regulation of pH and the production of lactic acid or acetic acid during ensilement in each treatment group. In addition, the correlations between propionic acid or butyric acid and bacteria in all samples indicated that *Weissella minor* and *Pediococcus pentosaceus* played essential roles in the production of propionic acid or butyric acid during the fermentation process. These results were consistent with the findings of previous studies, in which the abundance of *Lactiplantibacillus plantarum* was positively correlated with lactic acid concentration and negatively correlated with pH, indicating that the bacteria could grow at low pH levels and play an important role in ensiling fermentation process (Du et al., 2021b). Other studies also showed that the abundance of *Levilactobacillus brevis* was positively correlated with acetic acid concentration (Ding et al., 2020). After 60 days of ensiling, a high relative abundance of *Lactiplantibacillus plantarum* was observed in the SYK group, and the correlation between pH or lactic acid and microbes indicated that *Lactiplantibacillus plantarum* should be the major contributor to pH and lactic acid in SYK because *Lactiplantibacillus plantarum* is a homofermentative LAB that grows well in acidic environments and promotes lactic acid fermentation during ensiling (Du et al., 2022a). Furthermore, the pH is most closely related to the concentration of lactic acid (Kung et al., 2018). Considering the high relative abundance of *Levilactobacillus brevis* and the less relative abundance of *Lactiplantibacillus plantarum* in SSK, the major contributors to producing lactic acid and acetic acid in SSK could be *Lactiplantibacillus plantarum* and *Levilactobacillus brevis* because *Levilactobacillus brevis* is known as a heterofermentative strain belonging to the *Lentilactobacillus buchneri* group of lactobacilli (Daniel et al., 2015). In SCK, *Weissella minor*, *Latilactobacillus sakei*, *Levilactobacillus brevis* and *Lentilactobacillus buchneri* were the dominant bacteria, but the relative abundance of *Lactiplantibacillus plantarum* was low. Therefore, the SCK group had a higher pH and lower lactic acid concentration. *Lentilactobacillus buchneri* and *Levilactobacillus brevis* are known as heterofermentative LAB that produces acetic acid to increase the aerobic stability of silage (Du et al., 2021b). This was also the reason for the high acetic acid content in the SYK group. Wang et al. (2022a) reported that the abundance of *Weissella* was negatively correlated with pH after 3 days of ensilement, indicating that *Weissella* played an important role in reducing pH in the early stage, but *Weissella* was positively correlated with pH after 60 days of silage, which is similar to the resultes obtained in this study. The fermentation form of *Latilactobacillus sakei* during silage fermentation and the mechanism of its effect on ensiling are still unclear. Ding et al. (2020) reported that *Latilactobacillus sakei* was positively correlated with the concentrations of lactic acid and acetic acid, but in our study it was only found to be positively correlated with pH. We will continue to pay attention to the effect of *Latilactobacillus sakei* on silage fermentation in subsequent studies.

### Metabolic pathways and metabolic properties of ryegrass silage

Microorganisms promote ensiling fermentation by consuming substrates or transforming metabolites through complex metabolic pathways (Bai et al., 2021). At the same time, microbial metabolism affects the flavor and fermentation quality of silage (Du et al., 2022a). Metabolomics technology can more comprehensively display the metabolites in the microenvironment (Tian et al., 2022). Among the main differential metabolites of ryegrass silage at different growth stages, D-mannitol belongs to carbohydrates according to KEGG compound classification. Acetylcholine is a neurotransmitters; guanine, cytosine, deoxyguanosine, thymine, uridine belong to nucleic acids; citric acid and malic acid are organic acids; L-asparagine, N-formylmethionine, L-arginine, L-glutamine belong to amino acids, and D-Ala-D-Ala is a peptides. In the KEGG pathway, the amino acid metabolic pathway had the most metabolites, followed by the biosynthesis of other secondary metabolites, carbohydrate metabolism and nucleotide metabolism, which is consistent with the results of main differential metabolites of ryegrass silage, indicating that these pathways are of key importance for microbial metabolism. Xu et al. (2021) found that the metabolic pathways related to silage fermentation were the metabolism of carbohydrates, amino acid, energy and cofactors, and vitamins. Moreover, Du et al. (2021b) reported that carbohydrate metabolism and amino acid metabolism are the main microbial metabolic pathways affecting the flavor and quality of silage due to fatty acid metabolism, glycolysis and proteolysis. Our study supported this conclusion, as during the silage fermentation process of Italian ryegrass at different growth stages, the related important metabolic pathways were amino acid metabolism, synthesis of secondary metabolites, carbohydrate metabolism and nucleotide metabolism. Amino acids are primary metabolites produced by microorganisms through metabolic activities. They play an important role in plant protein synthesis and primary metabolism and are essential substances in plants (Du et al., 2021a). Carbohydrate metabolism mainly included glycolysis and gluconeogenesis (Kanehise and Goto, 2000), which provides energy for the life activities of LAB during fermentation. Nucleic acids are substrates of DNA synthesis and the main energy donor of cellular processes (Kilstrup et al., 2005). These metabolic pathways all promote the process of ensiling fermentation. The use of metabolomics technology not only enriched our understanding of silage metabolites, but also helped to understand the effects of growth stages on Italian ryegrass silage metabolic pathways, thus contributing to an in-depth understanding of silage processes and fermentation mechanisms (Fu et al., 2022). In addition, our study showed that phenylalanine, tyrosine and tryptophan biosynthesis was significantly enriched at booting stage; with the extension of growth period, the metabolic pathways significantly enriched in initial flowering stage increased, in which purine metabolism and ABC transporters pathway changed most obviously. The metabolic pathways significantly enriched at full flowering stage were similar to those at booting stage, but lack phenylpropanoid biosynthesis. The exact reason for this difference is still unclear, which may be related to the physiological process of forage raw materials in the growth process, or may be caused by microbial activity in the fermentation process. In the follow-up study, we will focus on it.

### Correlations between the relative abundances of bacteria and metabolites

In this study, metabolomics was used to analyze the differential metabolites, and Spearman correlation analysis was used to explore the correlations between the main fermentation bacteria and the main metabolites. After fermentation, the same metabolites were positively correlated with some lactic acid bacteria but negatively correlated with other species of lactic acid bacteria, indicating that there was competition and synergy between different species of bacteria during the ensiling fermentation process (Xu et al., 2018). After fermentation, cinnamic acid, 2-Hydroxycinnamic acid and uridine were positively correlate with *Latilactobacillus sakei*. Cinnamic acid is a polyphenols, is one of the most abundant plant secondary metabolites, and is ubiquitous in various plants (e.g., cinnamon, grapes; Feng et al., 2022). Cinnamic acid is an unsaturated carboxylic acid. Cinnamic acid and its derivatives possess broad-spectrum biological properties including anti-inflammatory capacities, antibacterial, antiviral, antifungal and anticancer activities (Ye et al., 2021; Feng et al., 2022). 2-Hydroxycinnamic acid is a phenolic acid with antibacterial and antioxidant activities (Peng et al., 2022). Uridine is a pyrimidine nucleoside with a variety of biologically active functions. It is an important nutritional supplement for regulating the harmful toxicity of antiviral and anticancer drugs and can be applied in many fields, such as health care, drug manufacturing and the food industry (Fan et al., 2018). The use of antibiotics in animal feed has attracted more and more attention, and antibacterial activity is becoming increasingly important in livestock feeding (Li et al., 2022d). Therefore, it is of great significance to produce silage with antibacterial activity for livestock production, and *Latilactobacillus sakei* can be considered as a functional inoculant for the production of silage with antibacterial activity. In the present study, we found that phenylacetaldehyde was negatively correlated with *Latilactobacillus sakei*. As phenylacetaldehyde is a rose-like aromatic compound with a strong honey-rose pleasant aroma (Szudera-Konczal et al., 2020), silage may have a weaker rose aroma when *Latilactobacillus sakei* abundance is high. In addition, we found that xanthine was positively correlated with *Levilactobacillus brevis*. Xanthine is the most ubiquitous heterocyclic aromatic compound, and the biological effects of xanthine and its derivatives, such as gustalgia, anti-inflammatory and diuretic effects, have been fully proved, and xanthine can be used as an antibacterial agents (Kapri et al., 2022). (E)-2-Methylglutaconic acid is a new isoleucine metabolite, and it may well be one of the key metabolites in the diagnosis of some future patients with ß-ketothiolase deficiency, propionic acidaemia or methylmalonic acidaemia (Duran et al., 1982). Since (E)-2-Methylglutaconic acid and *Lactiplantibacillus plantarum* were positively correlated, this may provide additional benefits for animal health. Tian et al. (2022) reported that malic acid had significant effects on the functions of bacterial communities. Malic acid has antioxidant and antibacterial functions, and has been widely used in food, pharmaceutical, health care and other industries. In addition, malic acid can be used as a source of carbohydrates to provide energy for the growth of LAB (Li et al., 2020). Malic acid is an important part of the TCA cycle, which directly regulates carbohydrate and protein metabolism (Li et al., 2020). According to the correlation analysis, bacteria associated with malic acid included *Lactiplantibacillus plantarum* and *Weissella minor*, indicating that these microorganisms might be potential targets for regulating carbohydrate metabolism during ensiling (Tian et al., 2022). Ganoderic acid F and D-Mannose were positively correlated with *Weissella minor*. As a natural bioactive monosaccharide, D-Mannose is a popular nutritional and health-beneficial food supplement all over the world, and it can be obtained from both plants and microorganisms (Hu et al., 2016). D-Mannose is an important component of polysaccharides and glycoproteins. It has been widely used in the food, pharmaceutical, and poultry industries, acting as the source of dietary supplements, as starting material for the synthesis of drugs and blocking colonization in animal feeds (Hu et al., 2016). In addition, Ganoderic acid F is an important triterpenoid compound and it has been reported to actively inhibit Matrigel-induced angiogenesis *in vivo*, suppress carcinogenesis and Epstein–Barr virus activation as an angiotensin-I-converting enzyme (ACE) inhibitor and has been used for the treatment of hypertension (Biswal et al., 2022). Therefore, *Weissella minor* can be considered as a feed inoculant with health care function in the production of silage.

Farnesyl acetone is an odor-related compound with sweet and green flue-cured tobacco flavors (Yao et al., 2022), and it was negatively correlate with *Weissella minor*, indicating that *Weissella minor* may affect the flavor of silage.

It is worth noting that although the results of correlation analysis based on statistics and related parameters cannot be considered causal (Tian et al., 2022), understanding the correlation between fermentation bacteria and metabolites can provide a theoretical reference for screening target lactic acid bacteria for high-quality silage, which is conducive to improving the production of animal products, providing higher welfare for animals, and improving livestock feed utilization, milk production, meat quality, and so on. In addition, we also hope to screen and apply silage inoculants with biomass secretion by metabolomics, such as amino acids, flavor agents and other bacteriostatic agents. This not only provides key information for the production of functional silage, but also will be a new field and new hot spot for future silage research (Xu et al., 2018).

## Conclusion

The growth stages had significant effects on the fermentation characteristics, bacterial community and metabolomics characteristics of Italian ryegrass silage. The silage of Italian ryegrass at booting stage had the best quality, the lowest pH value and the highest abundance of *Lactiplantibacillus plantarum*. The metabolic pathways at different growth stages were mainly concentrated in amino acid metabolism, followed by the biosynthesis of other secondary metabolites, carbohydrate metabolism and nucleotide metabolism, among which the amino acid metabolic pathway annotated the largest number of metabolites. Phenylalanine, tyrosine and tryptophan biosynthesis were significant at booting stage and full flowering stage; purine metabolism and ABC transporters pathway were more significant at initial flowering stage. *Latilactobacillus sakei* and *Weissella minor* have strong positive correlations with cinnamic acid and malic acid respectively, which have antibacterial and anti-inflammatory biological functions. This provides important information for screening functional LAB inoculants in the future.

## Data availability statement

The datasets generated for this study can be found in the NCBI under accession number SRP384413.

## Author contributions

ZF contributed to methodology, visualization, validation, and data curation and wrote the original draft. LS and QL interpreted the data and edited the language. YJ and ZW contributed to conceptualization, acquisition, reviewing, and editing. MH, JL, and JH contributed to software. GG contributed to conceptualization and funding acquisition. All authors contributed to the article and approved the submitted version.

## Funding

This work was supported by the Key Laboratory of Forage Cultivation and the Processing and Highly Efficient Utilization of the Ministry of Agriculture, the Key Laboratory of Grassland Resources of the Ministry of Education, and funded by the National Technical System of Forage Industry for Dry Grass Storage (CARS-34), China.

## Conflict of interest

The authors declare that the research was conducted in the absence of any commercial or financial relationships that could be construed as a potential conflict of interest.

## Publisher’s note

All claims expressed in this article are solely those of the authors and do not necessarily represent those of their affiliated organizations, or those of the publisher, the editors and the reviewers. Any product that may be evaluated in this article, or claim that may be made by its manufacturer, is not guaranteed or endorsed by the publisher.

## Footnotes

^1^https://cloud.majorbio.com

^2^www.majorbio.com
